# WTConv–TimesNet for Road Icing State Classification with IWOA-Based Hyperparameter Optimization

**DOI:** 10.3390/s26102980

**Published:** 2026-05-09

**Authors:** Lingqiu Cui, Yuxun Ji, Lijuan Zhang, Handong Li

**Affiliations:** 1 Guizhou Communications Polytechnic University, Guiyang 551400, China; gcpucuilq@126.com (L.C.); gcpulilj@126.com (L.Z.); 2 Guizhou Provincial Key Laboratory of Intelligent Construction and Operation & Maintenance for Bridge and Tunnel Engineering in Mountainous Areas, Guiyang 551400, China; 3 College of Electrical Engineering, Guizhou University, Guiyang 550025, China; xdli1@gzu.edu.cn

**Keywords:** road icing, icing state classification, multivariate time series, TimesNet, wavelet transform convolution, hyperparameter optimization

## Abstract

**Highlights:**

**What are the main findings?**

- An improved TimesNet network integrated with wavelet transform convolution is proposed and validated on a real-world road-icing dataset collected in the field. Compared with the baseline TimesNet model, it achieves an improvement of more than 6%.
- By introducing a new nonlinear factor, an Improved Whale Optimization Algorithm is developed. Together with feature selection, a more compact feature subset is constructed to reduce input redundancy, improving training efficiency while also increasing accuracy.

**What are the implications of the main findings?**

- A complete and reproducible workflow is established, covering raw-data preprocessing, feature construction, feature selection, and model training and evaluation. This provides a solid technical basis for road-icing early warning and intelligent road-safety management.
- Compared with the conventional TimesNet network, the improved TimesNet is more sensitive to high-frequency dynamics and local abrupt changes. It can effectively capture signal components that play a key role in icing-state transitions, such as temperature-difference disturbances and heat-flux fluctuations.

**Abstract:**

Road icing is a complex and highly dynamic phenomenon that poses a serious risk to winter road safety. However, nonlinear evolution, multiscale temporal dependence, and rapid transitions between adjacent stages still make it difficult to accurately identify icing states from multivariate environmental time-series data. In this work, a road icing state classification model based on TimesNet is developed. We integrate wavelet transform convolution (WTConv) into the original TimesNet architecture to strengthen multiscale time–frequency feature extraction, enabling more effective capture of high-frequency dynamics and abrupt local variations. To address the hyperparameter sensitivity commonly observed in icing scenarios, an Improved Whale Optimization Algorithm (IWOA) is employed and uses Pearson correlation analysis to select informative and physically meaningful features from multi-source monitoring data. Experiments on a real-road dataset show that the proposed IWOA–TimesNet–WTConv model improves overall accuracy from 92.72% to 98.83% compared with the baseline TimesNet model. In addition, feature selection yields a further 1.04 percentage-point gain in overall accuracy and increases the Macro F1-score from 0.9691 to 0.9809, indicating reduced redundancy and more stable discrimination under transitional icing conditions. Overall, the proposed method provides a practical and effective data-driven solution for intelligent road icing monitoring and early warning in complex winter road environments.

## 1. Introduction

Road icing is a critical phenomenon frequently observed on asphalt pavements in winter, particularly in high-altitude regions. Its occurrence is governed by complex interactions between environmental factors, including atmospheric pressure, ambient temperature, humidity, and the pavement’s internal thermal field [1,2,3]. Once ice forms, the tire–pavement friction coefficient drops markedly, escalating the risk of braking and steering failures and contributing significantly to winter traffic accidents [4]. To this end, achieving accurate road icing state classification is paramount for enhancing road safety and enabling intelligent traffic management.

With the advancement of sensing technologies and the Internet of Things (IoT), road environment monitoring has transitioned toward high-frequency, continuous, and automated paradigms. IoT-based systems, integrating multi-source sensors with edge computing, facilitate 24/7 monitoring with high spatiotemporal resolution, providing a robust empirical foundation for icing modeling and early warning [5,6]. Nevertheless, icing evolution is characterized by high nonlinearity and multi-scale dynamics, where state transitions often occur abruptly. In field conditions, changes between adjacent icing stages can happen within narrow time windows with indistinct boundaries; consequently, conventional identification methods tend to misclassify samples during rapid weather fluctuations [7,8]. Threshold-based or simplified physics-based approaches, which rely on fixed heuristic rules and rigid prior assumptions, lack the robustness required for complex and volatile road weather conditions [9].

Consequently, data-driven machine learning and deep learning methods have been extensively explored for road meteorological analysis and surface condition recognition. Architectures such as Recurrent Neural Networks (RNNs), Long Short-Term Memory (LSTM) Networks, and Temporal Convolutional Networks (TCNs) have demonstrated potential in modeling temporal dependencies [10,11,12]. For instance, Hu et al. leveraged vehicle-to-everything (V2X) data for automated icy pavement detection, while Tabrizi et al. employed LSTMs for hourly road surface temperature forecasting [13,14]. Wang et al. further improved highway icing time prediction using deep learning approaches based on data from road sensors [15]. However, conventional models often have difficulty capturing long-term dependencies and complex periodic patterns simultaneously, particularly when strong periodicity coexists with high-frequency disturbances. Recent studies on black ice prediction and road surface condition prediction further show that intelligent modeling of winter road states remains an active and important research area [16,17]. Although Transformer-based architectures have been introduced to model long-range interactions, many of these models rely primarily on time-domain representations and do not include explicit mechanisms for periodic modeling. As a result, their ability to respond to high-frequency transients and local variations is limited, which may lead to unstable performance during rapid transitions between adjacent icing stages [18,19]. Therefore, improving time–frequency modeling is essential for robust classification under complex icing scenarios [20]. Wavelet transform provides a natural multiscale representation and is well-suited to characterizing localized transient variations in non-stationary signals. Previous studies have demonstrated the effectiveness of wavelet-based classification in visual information processing systems, while the WTConv framework further shows that wavelet-domain convolution can be effectively integrated into deep architectures to enhance multi-frequency feature extraction [21,22]. These findings provide strong support for introducing wavelet-based representations into road icing time-series classification.

TimesNet addresses these limitations by transforming 1D time series into 2D multi-period representations, effectively capturing intra- and inter-period dependencies through specialized convolutions [23]. Despite its efficacy, TimesNet’s backbone remains largely focused on the time domain, which may weaken its capacity to extract high-frequency dynamics and abrupt signals, such as sharp temperature drops or heat-flux spikes, that often trigger icing state shifts. During icing evolution, such high-frequency cues can be decisive for transitions between adjacent stages; insufficient modeling of these signals may cause confusion among neighboring classes.

To address these challenges, this study develops a WTConv-enhanced TimesNet framework for fine-grained road icing state classification. Rather than simply combining existing modules, the proposed framework redesigns the temporal feature extraction process of TimesNet by replacing the original Inception convolution with a wavelet-based operation and incorporating the discrete wavelet transform into multiscale time–frequency analysis. This architectural refinement strengthens the ability of the model to capture high-frequency dynamics and abrupt variations while preserving the periodic modeling advantages of TimesNet. In addition, road icing data involves strongly coupled multi-source variables and rapid transitions between adjacent stages, which makes model performance highly sensitive to hyperparameter settings. Standard tuning strategies and conventional metaheuristic methods often exhibit unstable convergence or limited search capability in this setting. To address this issue, this study improves the classical whale optimization algorithm by introducing a nonlinear convergence factor and an adaptive weight strategy [24,25]. The resulting Improved Whale Optimization Algorithm (IWOA) enables joint optimization of critical hyperparameters, thereby improving convergence stability and generalization. Finally, to reduce redundancy in multi-source environmental features, a feature selection strategy based on Pearson correlation analysis is employed to construct a compact and physically interpretable feature subset, thereby improving both computational efficiency and predictive performance [26].

The primary contributions of this work are summarized as follows:

- Architecture Integration and Adaptation: A WTConv-enhanced TimesNet architecture is developed for multivariate road icing time-series classification by redesigning the original Inception module with wavelet-based operations, thereby strengthening multiscale time–frequency feature extraction while preserving the periodic modeling capability of TimesNet.
- Algorithmic Optimization: An Improved Whale Optimization Algorithm (IWOA) has been developed for robust hyperparameter optimization in road icing state recognition by introducing a nonlinear convergence factor and an adaptive weight strategy, thereby improving global search ability, convergence stability, and overall classification performance.
- Feature Refinement: A physically interpretable feature selection strategy is adopted based on Pearson correlation analysis to reduce data redundancy while preserving the predictive value of critical environmental variables.

Empirical Validation: Extensive experiments on real-world monitoring data show that the proposed framework achieves higher accuracy and better discrimination among adjacent icing states than representative baseline models.

The remainder of this paper is organized as follows. Section 2 describes the WTConv-enhanced TimesNet architecture and the IWOA. Section 3 details the dataset, preprocessing pipeline, and feature selection. Section 4 presents the experimental results, including hyperparameter optimization, ablation studies, and comparative analyses. Section 5 concludes the paper.

## 2. Methodology

### 2.1. Overall Framework of the Proposed Method

This study proposes an IWOA-TimesNet-WTConv framework for road icing state classification. By integrating WTConv into the TimesNet backbone, the framework improves multiscale time–frequency feature extraction and enhances the characterization of high-frequency dynamics and localized abrupt variations.

**Figure 1:**
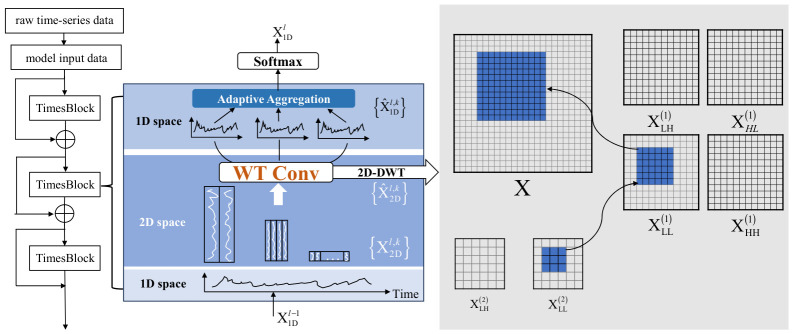
Overall framework of the proposed IWOA-optimized WTConv-TimesNet for road icing state classification.

The overall framework of the proposed method is illustrated in Figure 1. First, the raw time-series data are preprocessed and converted into model inputs using a sliding-window strategy. The resulting samples are then fed into the TimesNet backbone, where stacked TimesBlocks are employed for temporal feature modeling. Within each TimesBlock, the input sequence is transformed from the 1D domain into the 2D domain, and the original Inception convolution is replaced with the WTConv module to perform multiscale time–frequency feature extraction in the transformed space. The extracted features are subsequently mapped back to the 1D domain and fused through the Adaptive Aggregation module. Finally, the resulting representation is passed to a Softmax classification layer to produce the predicted road icing state. To further clarify the architecture of the proposed framework, the layer-wise input and output shapes of the main modules are summarized in Table 1. This table provides a clearer description of the network structure and helps illustrate the implementation details of the proposed IWOA-TimesNet-WTConv model.

### 2.2. TimesNet-Based Temporal Modeling

TimesNet is a deep learning model explicitly designed to exploit periodic structures in time series and is well-suited for complex temporal data exhibiting multiscale periodicity. Its core idea is to identify dominant periods within the input sequence and reorganize the original one-dimensional time series into a two-dimensional representation of time segments × period phases, enabling the joint modeling of intra-period and inter-period dependencies via convolution operations.

Let the input multivariate time series be denoted as

(1) $$X={\left[x_{1},x_{2},\ldots ,x_{t}\right]}^{T}\in {\mathbb{R}}^{\left(T\times F\right)}$$

where $x_{t}\in {\mathbb{R}}^{F}$ denotes the multivariate observation at the t−th time step, T represents the time step length and F denotes the number of feature channels. Based on frequency-domain analysis, TimesNet selects the top-K dominant periods ${\left\{p_{k}\right\}}_{k=1}^{K}$ with the highest spectral energy and reconstructs the input sequence accordingly. For each period scale $P_{k}$, the sequence is reshaped into a three-dimensional tensor:

(2) $$X^{\left(k\right)}\in {\mathbb{R}}^{\left(L_{k}\times p_{k}\times F\right)},L_{k}=[\frac{T}{p_{k}}]$$

where K denotes the number of selected dominant periods, $k\in \left\{1,2,\ldots ,K\right\}$ denotes the index of the k−th period scale, $p_{k}$ is the corresponding dominant period length, $L_{k}$ is the number of segments after period-wise reshaping, and $X^{\left(k\right)}$ denotes the reshaped feature tensor at the k−th period scale.

This periodic reorganization aligns time steps sharing the same phase within each period, facilitating explicit modeling of periodic patterns. At each scale, two-dimensional convolution is applied to extract temporal features:

(3) $$H^{\left(k\right)}=\;\sigma (\mathrm{Conv}2D(X^{\left(k\right)};W^{\left(k\right)})),H^{\left(k\right)}\in {\mathbb{R}}^{L_{k}\times p_{k}\times F}$$

where $H^{\left(k\right)}$ denotes the extracted feature tensor at the k−th period scale, $W^{\left(k\right)}$ denotes the learnable convolutional parameters corresponding to the k−th period scale, and σ(·) denotes the nonlinear activation function. The two-dimensional convolution operation can simultaneously capture local temporal patterns within the period and structural dependencies across periods.

Since feature tensors at different period scales vary in temporal length, resampling and pooling operations are employed to map them to a unified temporal dimension. The resulting multi-scale features are then concatenated and linearly projected to achieve multi-period information fusion. To stabilize network training and preserve original temporal information, a residual connection is introduced after the multi-period convolution module.

For road icing state classification, global pooling is applied to the extracted temporal features, followed by a fully connected layer and a Softmax function to produce the final class probabilities.

### 2.3. Wavelet Transform Convolution (WTConv)

Although TimesNet is effective at modeling periodic temporal structures, its original convolution-based design has limited ability to capture high-frequency dynamics and abrupt variations caused by environmental disturbances during road icing processes. To address this limitation, WTConv is integrated into the TimesNet architecture by replacing the original Inception convolution module, thereby strengthening multiscale time–frequency representation for multivariate road icing time-series classification.

Let the reshaped feature tensor at the k−th period scale be denoted as

(4) $$X^{\left(k\right)}\in {\mathbb{R}}^{L_{k}\times p_{k}\times F}$$

WTConv first applies a discrete wavelet transform (DWT) with the Daubechies wavelet basis to decompose the reshaped feature tensor into one low-frequency approximation sub-band and three high-frequency detail sub-bands:

(5) $$WT(X^{\left(k\right)})=\{{X^{\left(k\right)}}_{LL},{X^{\left(k\right)}}_{LH},{X^{\left(k\right)}}_{HL},{X^{\left(k\right)}}_{HH}\}$$

Here, ${X^{\left(k\right)}}_{\mathrm{LL}}$ denotes the low-frequency approximation sub-band, which captures the global trend of the reshaped feature tensor, whereas ${X^{\left(k\right)}}_{LH}$, ${X^{\left(k\right)}}_{HL}$, and ${X^{\left(k\right)}}_{HH}$ denote high-frequency detail sub-bands that characterize local abrupt changes, high-frequency perturbations, and fine-grained variations. The Daubechies wavelet basis has favorable orthogonality and compact support properties, which are beneficial for the multiscale decomposition of non-stationary signals and for preserving localized transient information across different frequency bands.

The decomposed sub-bands are concatenated along the channel dimension, and convolution operations are performed in the wavelet domain:

(6) $$H^{\left(k\right)}=\mathrm{Conv}\left({{\mathrm{Concat}(X}^{\left(k\right)}}_{LL}{X^{\left(k\right)}}_{LH},{X^{\left(k\right)}}_{HL},{X^{\left(k\right)}}_{HH});W\right),H^{\left(k\right)}\in {\mathbb{R}}^{L_{k}\times p_{k}\times F}$$

where W denotes the learnable convolution kernel parameters in the wavelet domain. Subsequently, an inverse wavelet transform is applied to recombine the processed frequency components and reconstruct the reshaped feature representation at the current period scale:

(7) $${\hat{X}}^{\left(k\right)}=\mathrm{IDWT}\left({X^{\left(k\right)}}_{LL},{X^{\left(k\right)}}_{LH},{X^{\left(k\right)}}_{HL},{X^{\left(k\right)}}_{HH}\right),\hat{X}\in {\mathbb{R}}^{L_{k}\times p_{k}\times F}$$

where ${\hat{X}}^{\left(k\right)}$ denotes the reconstructed output feature tensor of the WTConv module at the k−th period scale.

By performing convolution in the wavelet domain, WTConv enables more effective modeling of high-frequency dynamic features while preserving global trend information. When embedded into the TimesNet backbone, this design strengthens the representation of abrupt transitions and disturbance patterns across different period scales, thereby improving the ability of the model to characterize high-frequency variations in road icing processes.

### 2.4. Improved Whale Optimization Algorithm (IWOA)

To further enhance the stability and generalization performance of the WTConv–TimesNet model for road icing state classification, this study proposes an Improved Whale Optimization Algorithm (IWOA) to jointly optimize key model hyperparameters.

In road icing state recognition scenarios, multi-source environmental variables exhibit strong nonlinear coupling and rapid temporal variations. Model performance is therefore highly sensitive to hyperparameters such as network depth, hidden dimension, convolution kernel number, and wavelet decomposition levels. Improper parameter configurations may lead to unstable convergence and misclassification, particularly during transitional icing stages where boundaries between adjacent states are subtle and rapidly evolving. Conventional hyperparameter tuning strategies based on empirical selection or grid search are often less efficient and may be less suitable for such complex optimization tasks.

To address these limitations, the adopted IWOA framework introduces a nonlinear convergence factor and an adaptive weight strategy into the conventional WOA framework, drawing on representative improvement strategies reported in previous WOA studies [27,28]. The nonlinear convergence mechanism maintains stronger exploration capability in early iterations while enabling faster convergence in later stages, whereas the adaptive weight strategy dynamically balances global exploration and local exploitation. Together, these improvements enhance optimization stability, reduce the risk of premature convergence, and improve the ability of the algorithm to escape local optima during hyperparameter tuning for icing prediction tasks.

Assume a whale population of size N searching in a d-dimensional hyperparameter space. The position of the i−th whale is denoted as

(8) $$X_{i}=(x_{i}^{1},x_{i}^{2},\ldots ,x_{i}^{d})$$

and the position of the prey (current best solution) is represented by $X_{p}\left(t\right)$. During the encircling prey phase, the position update is defined as

(9) $$D=\left|C\cdot X_{p}\left(t\right)-X_{i}\left(t\right)\right|$$

(10) $$X\left(t+1\right)=X_{p}\left(t\right)-A\cdot D$$

where $X_{p}\left(t\right)$ denotes the current best solution, D represents the distance between the current whale and the best solution, and A and C are coefficient vectors used to control the search step and direction.

To enhance adaptability across different search stages, a nonlinear convergence factor is introduced to regulate A, defined as

(11) $$\left\{\begin{array}{l} A=2a\cdot r_{1}-a\\ C=2\cdot r_{2} \end{array}\right.$$

where $r_{1}$ and $r_{2}$ are random numbers uniformly distributed in [0, 1], and a is the nonlinear convergence factor defined as

(12) $$a=2-2\sin\left(\mu \frac{t}{m}\pi +\varphi \right)$$

where t denotes the current iteration number, m controls the nonlinearity of the convergence process, and μ and φ are constants used to regulate the shape of the nonlinear convergence curve.

During the exploitation phase, IWOA employs an adaptive weight mechanism to simulate the spiral updating behavior of whales:

(13) $$\left\{\begin{array}{l} \omega =1-\frac{e\frac{t}{m}-1}{e-1}\\ X\left(t+1\right)=\omega \cdot X_{p}\left(t\right)-A\cdot D \end{array}\right.$$

(14) $$X\left(t+1\right)=\omega \cdot X_{p}\left(t\right)+\left|X_{p}(t)-X(t)\right|\cdot e^{bl}\cdot \cos\left(2\pi l\right)$$

where ω denotes the adaptive weight, e denotes Euler’s number, b is a constant used to control the shape of the logarithmic spiral, l∈[−1,1] is a random number, and p∈[0,1] is used to switch between different position updating strategies. The mathematical model is given by

(15) $$X\left(t+1\right)=\left\{\begin{array}{cc} \omega \cdot X_{p}\left(t\right)-A\cdot D & p<0.5\\ \omega \cdot X_{p}\left(t\right)+\left|C\cdot X_{p}(t)-X(t)\right|\cdot e^{bl}\cdot \cos\left(2\pi l\right) & p\geq 0.5 \end{array}\right.$$

where $X_{\mathrm{rand}}\left(t\right)$ denotes the position of a randomly selected whale in the current population. To reduce the tendency of the algorithm to become trapped in local optima, IWOA uses $X_{\mathrm{rand}}\left(t\right)$ during the search phase, thereby increasing population diversity and enhancing global exploration of the search space. The expression is as follows:

(16) $$\left\{\begin{array}{l} D=\left|C\cdot X_{\mathrm{rand}}\left(t\right)-X\left(t\right)\right|\\ X\left(t+1\right)=X_{\mathrm{rand}}\left(t\right)-A\cdot D \end{array}\right.$$

Compared with the conventional WOA, the adopted IWOA exhibits stronger global exploration capability in the early optimization stage and achieves more stable convergence in later iterations, which helps alleviate premature convergence during hyperparameter optimization.

## 3. Data Preparation and Evaluation Metrics

### 3.1. Data Preprocessing and Sample Construction

This study uses multi-source time-series data collected from an in situ road environmental monitoring system to train and evaluate the proposed road icing state classification model. The raw data are recorded at fixed time intervals and include ambient temperature, pavement surface temperature, NTC temperature (i.e., contact-measured pavement temperature obtained using a negative temperature coefficient thermistor), relative humidity, barometric pressure, barometric temperature, wind speed, wind direction, illumination intensity, and wind speed sensor output voltage. Illumination variation is included in the model as one of the monitored environmental variables and is learned jointly with the other sensor measurements. These data are multivariate, have high temporal resolution, and exhibit strong temporal dependence.

**Figure 2:**
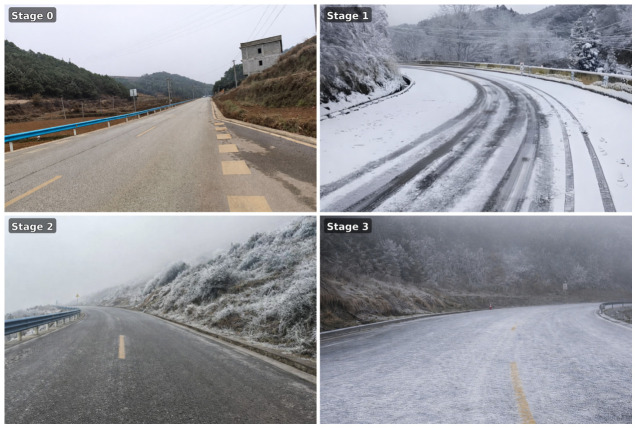
Representative examples of road icing states used for labeling: Stage 0 (non-icing), Stage 1 (mild icing), Stage 2 (moderate icing), and Stage 3 (severe icing).

Based on pavement thermodynamic behavior and the evolution characteristics of road icing processes, road conditions are categorized into four discrete icing states: non-icing, mild icing, moderate icing, and severe icing, denoted as Stage 0 to Stage 3, respectively. Representative examples of the four stages used for label definition are presented in Figure 2, which provides an intuitive interpretation of the target classes for supervised learning.

To transform raw sensor logs into supervised learning samples, the preprocessing and sample construction procedure is conducted sequentially as follows. First, raw logs from heterogeneous sensors are parsed and temporally aligned using timestamps to ensure cross-sensor synchronization. To mitigate sensor latency and occasional sampling inconsistencies, a time-window-based alignment strategy is adopted. For angular variables such as wind direction, sine transformation is applied to eliminate artificial discontinuities caused by angular wrap-around. Missing values occurring sporadically are handled using forward and backward filling to preserve temporal continuity.

Next, feature construction is performed to enhance physical interpretability and dynamic representation. Temperature measurements from multiple sensors are fused to form a comprehensive air temperature feature, reducing the influence of individual sensor noise. Two temperature-difference features are then introduced—between pavement surface temperature and air temperature, and between NTC temperature and air temperature—to characterize pavement–atmosphere heat exchange. Furthermore, to capture short-term dynamics, first-order differences (variation rates), moving averages, and moving standard deviations of the temperature-difference series are computed using sliding windows, enabling the model to better represent local trends and fluctuations.

Finally, all continuous features are normalized to the range $\left[0,1\right]$ using Min–Max scaling to eliminate the influence of differing physical units. Supervised samples are then generated using a sliding window strategy. Given a window length T and step size S, each sample consists of multivariate features from T consecutive time steps, and the corresponding label is determined by the road icing stage at the prediction horizon. Through this procedure, continuous multi-source time-series logs are converted into structured samples suitable for training and evaluating the proposed classification model.

### 3.2. Feature Selection

In multi-source environmental monitoring data, the relevance of different variables to road icing states varies considerably. Direct use of all candidate features may introduce redundancy and multicollinearity, which can negatively affect model stability and generalization. Therefore, Pearson correlation coefficient (PCC) analysis is employed to quantify the linear association between candidate features and road icing state labels. PCC values are computed for all candidate features, and statistical significance is assessed using *p*-values. The resulting correlation statistics are summarized in Table 2.

**Figure 3:**
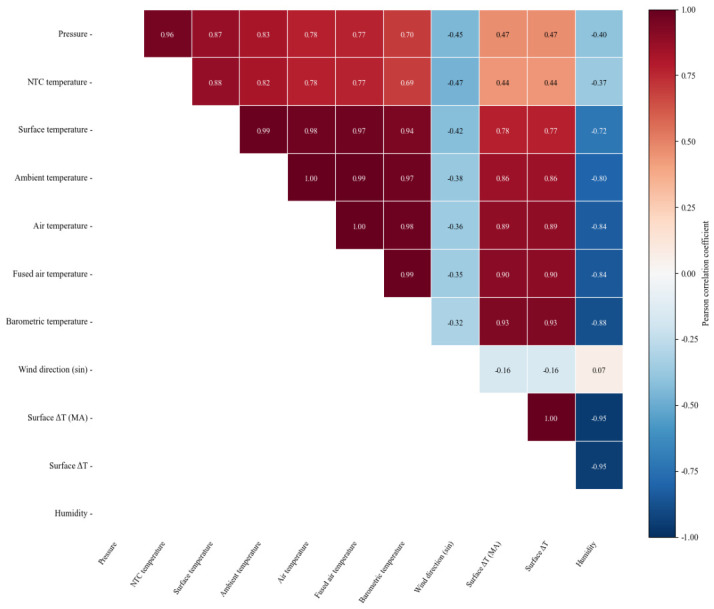
Pearson correlation heatmap among selected features.

The results show that pressure-related variables, NTC temperature, pavement surface temperature, and ambient temperature are strongly correlated with road icing states, highlighting the dominant role of thermodynamic factors in icing formation and evolution. To further examine dependencies among the selected features, pairwise PCC values are computed and visualized in the correlation heatmap shown in Figure 3; the color values represent Pearson correlation coefficients ranging from −1 to 1, where positive values indicate positive correlations, negative values indicate negative correlations, and larger absolute values indicate stronger relationships. However, a strong correlation does not necessarily imply a direct causal relationship. For example, in the present dataset, the correlation between pressure and NTC temperature is more reasonably interpreted as a joint response to shared winter meteorological conditions and surface thermal states during icing-prone periods. Under these conditions, atmospheric pressure and pavement temperature measured by the NTC sensor tend to vary in opposite directions, resulting in a pronounced negative correlation between the two variables. Strong correlations are also observed among temperature-related variables, whereas temperature-difference features show relatively weaker correlations with raw temperature measurements, suggesting that they provide complementary information. Based on these analyses, a compact feature set with clear physical interpretability is retained as input to the subsequent model.

### 3.3. Evaluation Metrics

To comprehensively evaluate model performance in road icing state classification, both overall accuracy and class-wise discrimination ability are considered. Overall performance is measured using Accuracy, whereas Precision, Recall, and F1-score are used to assess classification performance for each icing state. To provide an overall evaluation across multiple classes, macro-averaged Precision, Recall, and F1-score are calculated by averaging the corresponding class-wise metrics. In addition, confusion matrices are used to analyze misclassification patterns among different icing states, allowing intuitive assessment of the ability of the model to distinguish adjacent icing stages.

## 4. Experimental Results and Discussion

All experiments were conducted on a workstation equipped with an Intel Core i5-12600 K CPU (Intel, Santa Clara, CA, USA), an NVIDIA GeForce RTX 3080 Ti GPU (Nvidia, Santa Clara, CA, USA) with 12 GB of memory, and 32 GB of RAM. The software environment consisted of Python 3.8.5, PyTorch 2.2.0, and CUDA 11.8 for GPU acceleration. To ensure fairness and comparability, the proposed model and all baseline models were trained and evaluated under identical hardware and software conditions.

### 4.1. Hyperparameter Optimization Results

The WTConv-TimesNet model involves multiple architecture-related and training-related hyperparameters, and their configurations have a substantial effect on classification performance. To avoid subjective manual tuning and reduce the risk of convergence to local optima, the Improved Whale Optimization Algorithm (IWOA) is employed to jointly optimize the key hyperparameters. The optimization procedure is implemented in Python using PyTorch with a self-developed search routine. Validation loss is selected as the optimization objective because it provides a smoother and more sensitive signal than accuracy during hyperparameter search, especially when accuracy remains unchanged across different candidate configurations in the early stages of training.

**Figure 4:**
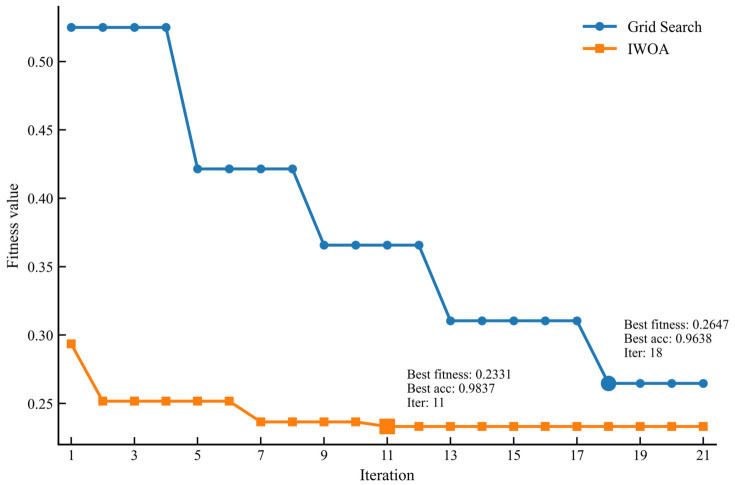
Fitness evolution comparison between IWOA and grid search during hyperparameter optimization.

Figure 4 compares the fitness evolution of IWOA and conventional grid search during hyperparameter optimization. As shown in the figure, the fitness value of IWOA decreases rapidly in the early iterations and stabilizes at a lower level in the later stages, indicating an efficient and stable search process in the hyperparameter space. In contrast, grid search improves more slowly and converges to a higher fitness value. Specifically, IWOA achieves a lower best fitness value than grid search (0.2331 vs. 0.2647) and yields higher classification accuracy (0.9837 vs. 0.9638). In addition, IWOA reaches its best fitness value at the 11th iteration, whereas grid search reaches its best result at the 18th iteration. These results demonstrate the effectiveness of IWOA for hyperparameter optimization and further indicate that it is more efficient than conventional grid search in this task.

The optimal hyperparameter configuration identified by IWOA is summarized in Table 3. Under this configuration, the proposed model attains its best validation performance, providing a reliable basis for the subsequent experiments.

These results confirm that the nonlinear convergence factor and adaptive weighting strategy introduced in IWOA effectively balance global exploration and local exploitation, making it well-suited for hyperparameter-sensitive road icing recognition tasks.

### 4.2. Ablation Study

To systematically evaluate the effectiveness of each proposed component, ablation experiments were conducted by progressively introducing WTConv and IWOA into the baseline TimesNet model. The following three models were compared:

- TimesNet (baseline);
- TimesNet–WTConv;
- IWOA–TimesNet–WTConv (proposed).

Table 4 reports the quantitative performance of different ablation models on the test set. Compared with the baseline TimesNet, the introduction of WTConv improves overall accuracy from 92.72% to 95.97%, corresponding to a relative increase of 3.5%, while the macro-averaged F1-score increases from 0.8757 to 0.9316, yielding an improvement of approximately 6.4%. This demonstrates that the wavelet-based convolution effectively enhances the model’s ability to capture multiscale temporal patterns and high-frequency dynamics associated with icing evolution.

After further integrating IWOA-based hyperparameter optimization, the proposed IWOA–TimesNet–WTConv model achieves an overall accuracy of 98.83%, representing an additional 2.86% absolute improvement over the WTConv-enhanced model and a 6.11% improvement over the baseline TimesNet. Similarly, the Macro F1-score increases to 0.9806, which is 10.5% higher than that of the baseline model.

**Figure 5:**
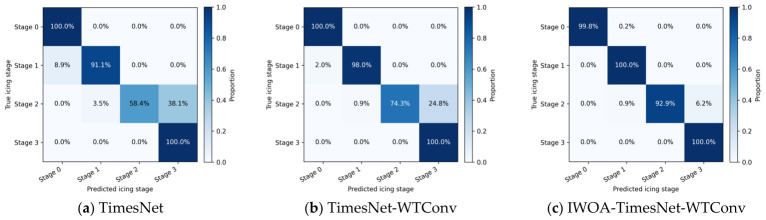
Confusion matrix comparison of different ablation models for road icing stage classification.

Figure 5 presents the confusion matrices of the three ablation models. The baseline TimesNet exhibits noticeable confusion between adjacent icing stages, particularly between mild and moderate icing. The incorporation of WTConv substantially reduces these misclassifications, while the fully optimized model further sharpens decision boundaries, resulting in more concentrated diagonal distributions. This indicates a significantly improved capability in distinguishing closely related icing states.

### 4.3. Effectiveness of Feature Selection

**Figure 6:**
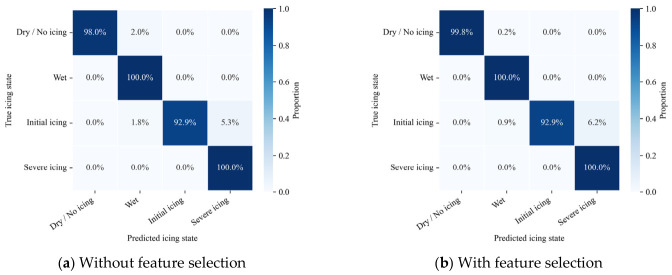
Confusion matrices of road icing state classification results: (**a**) model performance without feature selection, showing higher misclassification between adjacent icing stages; (**b**) model performance with feature selection, where misclassification is reduced, and classification boundaries become clearer, demonstrating improved discrimination among icing states.

To evaluate the effectiveness of the proposed feature selection strategy, a comparative experiment was conducted by training and testing the model with and without feature selection, while keeping the network architecture and training settings unchanged. The corresponding quantitative results are summarized in Table 5, and the confusion matrices are illustrated in Figure 6.

With feature selection, the overall accuracy increases from 97.79% to 98.83%, corresponding to an improvement of 1.04 percentage points, while the Macro F1-score improves from 0.9691 to 0.9809, yielding a relative gain of approximately 1.2%. Although the numerical improvement appears moderate, feature selection substantially reduces input redundancy and improves classification stability, especially for non-icing and early icing states.

The confusion matrices in Figure 6 further reveal the impact of feature selection on class-wise discrimination. After removing redundant and weakly correlated features, misclassifications between adjacent icing states are noticeably reduced. In particular, the confusion between non-icing and mild icing states is significantly alleviated, with predictions becoming more concentrated along the diagonal. This observation suggests that feature selection contributes to a clearer decision boundary and improved robustness in challenging transitional states.

From an engineering perspective, the proposed feature selection strategy not only reduces input dimensionality and mitigates the negative effects of feature redundancy but also enhances the utilization efficiency of physically meaningful environmental variables. By retaining features closely related to the thermodynamic mechanisms of road icing, the model becomes more sensitive to icing evolution while maintaining computational efficiency. These results demonstrate that the adopted feature selection strategy effectively improves both classification accuracy and practical applicability of the road icing state recognition system.

### 4.4. Comparison with Other Models

To further validate the superiority of the proposed approach, the IWOA–TimesNet–WTConv model was compared with several representative time-series classification methods, including BiLSTM, TCN, Transformer, and TSMixer. All models were trained and evaluated under identical data partitions, input features, and evaluation metrics to ensure fairness.

As shown in Table 6, the proposed IWOA-TimesNet-WTConv model achieves the best classification performance among all compared methods, with an overall accuracy of 98.83%, a Macro Precision of 0.9807, a Macro Recall of 0.9817, and a Macro F1-score of 0.9806. In terms of overall accuracy, it outperforms BiLSTM, TCN, Transformer, and TSMixer by 11.57, 7.15, 5.20, and 7.02 percentage points, respectively. Its macro F1-score is also higher than those of BiLSTM, TCN, Transformer, and TSMixer by 0.1889, 0.1208, 0.0862, and 0.1190, respectively, indicating stronger robustness in distinguishing different road icing states.

From the perspective of computational cost, the proposed model has 20,804 total parameters and 16,708 trainable parameters, with a computational cost of 513.792 K FLOPs. Its computational burden is substantially lower than that of BiLSTM and Transformer, while remaining moderately higher than that of TCN and TSMixer. Specifically, compared with BiLSTM and Transformer, the proposed model reduces FLOPs by approximately 54.5% and 91.6%, respectively. Although TCN and TSMixer are lighter in computational cost, their classification performance is clearly inferior. These results indicate that the proposed model achieves a favorable balance between classification accuracy and computational efficiency.

These findings indicate that conventional recurrent and convolutional models have limited ability to simultaneously capture long-term dependencies, periodic structures, and high-frequency disturbances in road icing processes. Although Transformer-based models improve global dependency modeling, their sensitivity to local high-frequency and non-stationary variations remains insufficient. By contrast, the proposed model effectively integrates periodic modeling, multiscale time–frequency feature extraction, and adaptive hyperparameter optimization, thereby enabling more robust and balanced classification across different road icing states.

### 4.5. Discussion and Limitations

From a quantitative perspective, the proposed IWOA-TimesNet-WTConv framework consistently exhibits superior performance compared to the baseline TimesNet model and other representative methodologies under equivalent experimental configurations. Nevertheless, several limitations warrant further investigation. Regarding computational overhead, although the proposed model is significantly more efficient than the BiLSTM and Transformer architectures, it remains more computationally demanding than the TCN and TSMixer models. This disparity suggests that the enhancement of computational efficiency while maintaining high classification accuracy remains a critical objective. Additionally, the current experimental evaluations are derived from data acquired from a geographically constrained road section under specific winter conditions. While the results establish the efficacy of the proposed framework on the current dataset, further validation across heterogeneous road environments, climatic conditions, and pavement types is required to assess the broader capability for generalization.

An additional limitation resides in the focus of the current framework on temporal feature modeling within multi-source monitoring sequences. Although this design effectively captures periodic patterns and high-frequency variations during icing evolution, the isolated impact of severe sensor disturbances and abnormal environmental fluctuations on the performance of the classification has not yet been quantitatively evaluated, despite the inclusion of illumination intensity as an input variable. Furthermore, potential spatial interactions among distinct monitoring locations are not explicitly addressed. In practical road monitoring scenarios, such spatial dependencies could provide valuable insights for enhancing the reliability of the classification. Consequently, future research will follow three primary trajectories: initially, more efficient architectural designs will be explored to reduce computational complexity and enhance deployment flexibility; subsequently, cross-scenario validation involving larger and more heterogeneous datasets will be conducted to bolster the robustness and generalization capability of the proposed method; and finally, spatiotemporal modeling strategies will be incorporated to better characterize both the temporal evolution and spatial heterogeneity of road icing processes, thereby further enhancing the applicability of the method in real-world monitoring and early warning tasks. From an operational standpoint, the current framework is well-suited for road monitoring scenarios characterized by the availability of multi-source environmental sensing and the requirement for reliable recognition of transitional icing states.

## 5. Conclusions

To address the difficulty of accurately identifying road icing states under complex environmental conditions, this study proposed an improved TimesNet-based framework, namely IWOA–TimesNet–WTConv, for road icing state classification. By integrating WTConv into the TimesNet backbone and introducing IWOA for hyperparameter optimization, the proposed method improves the representation of complex temporal patterns in multi-source road environmental data and enhances classification performance for four icing states.

Experiments on real-world multi-source road monitoring data demonstrate the effectiveness of the proposed framework. Compared with the baseline TimesNet model, the introduction of WTConv improves the overall classification accuracy from 92.72% to 95.97%. After further hyperparameter optimization using IWOA, the final model achieves an accuracy of 98.83%, corresponding to an overall improvement of 6.11 percentage points over the baseline. In addition, Macro Precision, Recall, and F1-score all reach approximately 0.98, indicating balanced and stable classification performance across different icing states. Feature selection based on Pearson correlation analysis further improves model performance from 97.79% to 98.83%, while also reducing redundant input features.

Overall, the proposed method provides an effective data-driven solution for road icing state classification and offers useful support for intelligent road icing monitoring and early warning. These findings demonstrate the potential of the proposed method for practical road icing monitoring and early warning applications.

## Figures and Tables

**Table 1:** Layer-wise input and output shapes of the proposed IWOA–TimesNet–WTConv model.

Layer	Input Shape	Output Shape
Input projection	(32, 30, 11)	(32, 30, 64)
FFT-based period discovery	(32, 30, 64)	Top−K periods = [30, 15, 10, 7, 6]
1D-to-2D reshaping	(32, 30, 64)	(32, 64, 1, 30) (32, 64, 2, 15) (32, 64, 3, 10) (32, 64, 5, 7) (32, 64, 6, 5)
WTConv	(32, 64, 1, 30) (32, 64, 2, 15) (32, 64, 3, 10) (32, 64, 5, 7) (32, 64, 6, 5)	(32, 64, 1, 30) (32, 64, 2, 15) (32, 64, 3, 10) (32, 64, 5, 7) (32, 64, 6, 5)
Adaptive aggregation (2D-to-1D)	(32, 64, 1, 30) (32, 64, 2, 15) (32, 64, 3, 10) (32, 64, 5, 7) (32, 64, 6, 5)	(32, 30, 64)
Residual connection	(32, 30, 64)	(32, 30, 64)
GELU	(32, 30, 64)	(32, 30, 64)
Dropout	(32, 30, 64)	(32, 30, 64)
Flatten	(32, 30, 64)	(32, 1920)
Fully connected layer	(32, 1920)	(32, 4)

**Table 2:** Pearson correlation statistics between candidate features and road icing states.

Feature	Pearson Correlation (r)	*p*-Value	Sample Size (n)	|r|
Pressure (avg)	−0.964	<1 × 10^−300^	58,355	0.964
NTC temperature (avg)	−0.950	<1 × 10^−300^	58,355	0.95
Surface temperature (avg)	−0.853	<1 × 10^−300^	58,355	0.853
Ambient temperature (avg)	−0.795	<1 × 10^−300^	58,355	0.795
Air temperature (avg)	−0.750	<1 × 10^−300^	58,355	0.75
Fused air temperature	−0.742	<1 × 10^−300^	58,355	0.742
Barometric temperature (avg)	−0.673	<1 × 10^−300^	58,355	0.673
Wind direction (sin)	0.462	<1 × 10^−300^	58,355	0.462
Surface ΔT	−0.417	<1 × 10^−300^	58,355	0.417
(moving average)				
Surface ΔT	−0.416	<1 × 10^−300^	58,355	0.416
Humidity (avg)	0.358	<1 × 10^−300^	58,355	0.358
NTC ΔT (moving std)	0.144	7.0 × 10^−267^	58,355	0.144
Wind speed (avg)	0.129	1.77 × 10^−213^	58,355	0.129
NTC ΔT	−0.118	2.42 × 10^−180^	58,355	0.118
(moving average)				
NTC ΔT	−0.118	2.92 × 10^−180^	58,355	0.118
Surface ΔT (moving std)	0.037	2.67 × 10^−19^	58,355	0.037
NTC ΔT change rate	−0.001	0.897	58,355	0.001
Surface ΔT change rate	−0.000	0.949	58,355	0

**Table 3:** Optimal hyperparameter configuration of the IWOA-optimized TimesNet–WTConv model.

Hyperparameter	Value
Number of encoder layers (e_layers)	2
Model dimension (d_model)	64
Feed-forward dimension (d_ff)	64
Number of dominant periods (top_k)	5
Number of convolution kernels (num_kernels)	3
Wavelet decomposition levels (wt_levels)	2
Dropout rate	0.05

**Table 4:** Performance comparison of ablation models for road icing state classification.

Model	Overall Accuracy	Macro Precision	Macro Recall	Macro F1-Score
TimesNet	0.9272	0.9161	0.8737	0.8757
TimesNet–WTConv	0.9597	0.9476	0.9309	0.9316
IWOA–TimesNet–WTConv	0.9883	0.9807	0.9812	0.9806

**Note:** Macro Precision, Macro Recall, and Macro F1-score are computed by averaging class-wise metrics across four road icing states.

**Table 5:** Performance comparison of the model with and without feature selection on the test set.

Model Setting	Overall Accuracy	Macro Precision	Macro Recall	Macro F1-Score
Without feature selection	0.9779	0.9655	0.9779	0.9691
With feature selection	0.9883	0.9802	0.9883	0.9809

**Table 6:** Performance and computational cost comparison of different models for road icing state classification.

Model	Overall Accuracy	Macro Precision	Macro Recall	Macro F1-Score	Total Parameters	Trainable Parameters	FLOPs
BiLSTM	0.8726	0.7865	0.8112	0.7917	36,868	36,868	1.129 M
TCN	0.9168	0.8919	0.8529	0.8598	18,180	18,180	203.392 K
Transformer	0.9363	0.9246	0.8887	0.8944	219,524	219,524	6.117 M
TSMixer	0.9181	0.9056	0.8567	0.8616	12,117	12,117	170.280 K
IWOA–TimesNet–WTConv	0.9883	0.9807	0.9817	0.9806	20,804	16,708	513.792 K

## Data Availability

Due to project data management restrictions, the data presented in this study are available from the corresponding author upon reasonable request. The full dataset cannot be made publicly available prior to formal publication. However, partial datasets necessary to verify the research findings can be provided upon reasonable request. The complete dataset will be made publicly available after the study is formally published.
